# Safety and efficacy of auricular acupuncture in patients with depression after percutaneous coronary intervention: A protocol for systematic review and meta-analysis

**DOI:** 10.1097/MD.0000000000029173

**Published:** 2022-04-15

**Authors:** Ruozhu Lu, Rui Shi, Miao Zhang, Xiao Shao, Wen Xue, Qian Guo, Cheng Wang, Yue Deng

**Affiliations:** a School of Traditional Chinese Medicine, Changchun University of Chinese Medicine, Changchun, China; b Branch of National Clinical Research Center for Chinese Medicine Cardiology, Hospital of Affiliated Changchun University of Chinese Medicine, Changchun, China; c School of Basic Medicine, Changchun University of Chinese Medicine, Changchun, China.

**Keywords:** auricular acupuncture, depression, percutaneous coronary intervention, protocol, systematic review

## Abstract

**Background:**

With the advantages of miniature damage and optimal effectiveness, percutaneous coronary intervention (PCI) has been performed in a large number of coronary artery disease patients. However, recent studies have indicated a higher incidence of depression on post-PCI patients. Acupuncture therapy is effective for depression. As a form of acupuncture, the auricular acupuncture has been used to relieve symptoms in patients with post-PCI depression, but its effectiveness and safety have not yet reached a definitive conclusion. Therefore, this systematic review and meta-analysis protocol is planned to evaluate the efficacy and safety of auricular acupuncture for depression in post-PCI patients.

**Methods:**

Six English databases (PubMed, Web of Science, MEDLINE, EMBASE, Springer Cochrane Library, and WHO International Clinical Trials Registry Platform) and 4 Chinese databases (Wan Fang Database, Chinese Scientific Journal Database, China National Knowledge Infrastructure Database, and Chinese Biomedical Literature Database) will be searched normatively according to the rule of each database from the inception to February 1, 2022. Two reviewers will independently conduct article selection, data collection, and risk of bias evaluation. Any disagreement will be resolved by discussion with the third reviewer. Either the fixed-effects or random-effects model will be used for data synthesis based on the heterogeneity test. The change in the scores on the Hamilton Depression Scale and the Self-rating Depression Scale will be used as the main outcome measure. All-cause mortality, cardiac mortality, major adverse cardiovascular events, rehospitalisation rate, and Quality of Life Scale as the secondary outcome. Treatment Emergent Symptom Scale, general physical examination (temperature, pulse, respiration, blood pressure), routine examination of blood, urine and stool, electrocardiogram, liver and kidney function examination as the security indexes. RevMan 5.3.5 will be used for meta-analysis.

**Results:**

This study will provide high-quality evidence to assess the efficacy and safety of auricular acupuncture for depression in post-PCI patients.

**Conclusion:**

This systematic review will explore whether auricular acupuncture is an effective and safe intervention for depression in post-PCI patients.

**INPLASY registration number:**

INPLASY202230003.

## 1 Introduction

Coronary heart disease has become a cardiovascular disease that seriously endangers human health. With the advantage of miniature damage and optimal effectiveness, percutaneous coronary intervention (PCI) has been performed in a high number of coronary artery disease patients.^[1]^ However, major adverse cardiovascular events (MACEs) still pose clinical problems after PCI. Known cardiac risk factors, such as hypercholesterolaemia and dyslipidemia have found to predict adverse cardiac events. Besides these well-known conventional risk factors, Psychological factors, such as depression and anxiety, might be important in predicting adverse outcomes after PCI.^[2]^ Many studies have demonstrated increased prevalence of depressive disorder in patients with MI or received PCI as well as poor outcomes in the long-term.^[3–6]^ Post-MI depression is an independent predictor of 3-year major acute cardiovascular events (MACEs), mortality, and repeat revascularization.^[7]^ A prospective longitudinal study which included 133 post-PCI patients indicated the probability of recurrent cardiac events in patients after PCI is positively correlated with anxiety and depression status.^[8]^ And a multicenter prospective study show that depression is associated with an increased risk for MACEs post-PCI, independent of anxiety.^[2]^ The results of a meta-analysis show that depressed individuals in post-PCI have a 57% greater risk of poor outcome than nondepressed patients.^[9]^

Post-PCI depression consists mainly of psychotherapy and antidepressant medications.^[10]^ Antidepressants have potential adverse effects such as sexual dysfunction, weight changes and sleep disruption. Drug interactions also need to be taken into consideration when the usage of antidepressants was combined with cardiovascular medicine. For example, various recent guidelines across the world had place selective serotonin reuptake inhibitors as the first line antidepressant^[11]^ while post-PCI patients often has to correspondingly undergo a guideline recommended 1-year regular intake of anticoagulants and dual antiplatelet therapy.^[12]^ Recent studies had correlated the combined medications of selective serotonin reuptake inhibitors, anticoagulants, and dual antiplatelet with a higher risk of hemorrhage.^[13,14]^ In addition, studies measuring treatment such as psychotherapy, exercise and collaborative care demonstrated effectiveness but the strength of the effects was weak in most studies, and no proven benefits in cardiovascular, all-cause morbidity and mortality. Therefore, we tried to find a complementary or alternative method to treat post-PCI depression.

Acupuncture is the world renowned ancient Chinese therapy which had long served China's medical system for more than 3000 years. The theories employed in acupunctures are derived from the concept of holism, viscera, and meridians in traditional Chinese medicine. Previous meta-analysis proved acupuncture had satisfactory results on both efficacy and safety in the treatment of depression.^[11]^ As a form of acupuncture, the auricular acupuncture has been used to relieve symptoms in patients with post-PCI depression, but its effectiveness and safety have not yet reached a definitive conclusion.^[15]^ Therefore, this research intends to adopt the method of system valuation and meta-analysis of the auricular acupuncture for post-PCI depression to evaluate the efficacy and safety.

## 2 Methods

### 2.1 Study registration

This protocol will be conducted under the preferred reporting items for systematic reviews and meta-analyses protocols guidelines.^[15]^ Furthermore, the study has been registered on International Platform of Registered Systematic Review and Meta-Analysis Protocols (https://inplasy.com/), registration number: INPLASY202230003.

### 2.2 Inclusion criteria for study selection

#### 2.2.1 Types of studies

All relevant randomized controlled trials (RCTs) in English and Chinese will be included. While non-RCTs, quasi-RCTs, cohort studies, reviews, case reports, experimental studies, expert experience, the data of the included study is missing or incomplete, and duplicate publications will be excluded to ensure the quality of this systematic review.

#### 2.2.2 Types of participants

Participants of different age groups with depression following PCI could be included in the study, regardless of nationality, race, gender, occupation, and educational background. While the cause of depression is not limited, experimental objects which included patients with schizophrenia would be excluded.

#### 2.2.3 Types of interventions

This study focuses on the RCTs of depression under the treatment of auricular acupuncture. The treatment group should be treated by auricular acupuncture combining or not combining with western medicines. The results are anticipated to aid clinicians. All trials with an assessment of the treatment mentioned above will be included, while studies of control group could only use western medicines as the sole treatment.

#### 2.2.4 Types of outcome measures

##### 2.2.4.1 Primary outcomes

The primary outcomes are the Hamilton Depression Scale and the Self-rating Depression Scale.

##### 2.2.4.2 Secondary outcomes

The secondary outcomes of this review mainly include the following aspects: all-cause mortality, cardiac mortality, MACEs, rehospitalization rate, and Quality of Life Scale.

##### 2.2.4.3 Security index

Treatment Emergent Symptom Scale.

General physical examination (temperature, pulse, respiration, blood pressure).

Routine examination of blood, urine and stool.

Electrocardiogram.

Liver and kidney function examination.

### 2.3 Data sources

Six English databases (PubMed, Web of science, Medline, EMBASE, Springer Cochrane Library, and WHO International Clinical Trials Registry Platform) and 4 Chinese databases (Wan fang Database, Chinese Scientific Journal Database, China National Knowledge Infrastructure Database, and Chinese Biomedical Literature Database) will be searched normatively in accordance with the rule of each database from the inception to February 1, 2022.

### 2.4 Searching strategy

Search strategy will be built in accordance with the guidelines from the Cochrane handbook. The Search strategy for PubMed is shown in Table 1, which included all search terms, and similar strategies will be built and applied for other electronic databases.

**Table 1:** The search strategy for PubMed.

Number	Search terms
#1	(“Depression”[Mesh]) OR ((((((Depressive Symptoms[Title/Abstract]) OR (Depressive Symptom[Title/Abstract])) OR (Symptom, Depressive[Title/Abstract])) OR (Symptoms, Depressive[Title/Abstract])) OR (Emotional Depression[Title/Abstract])) OR (Depression, Emotional[Title/Abstract]))
#2	(“Percutaneous Coronary Intervention”[Mesh]) OR (((((((((((Coronary Intervention, Percutaneous[Title/Abstract]) OR (Coronary Interventions, Percutaneous[Title/Abstract])) OR (Intervention, Percutaneous Coronary[Title/Abstract])) OR (Interventions, Percutaneous Coronary[Title/Abstract])) OR (Percutaneous Coronary Interventions[Title/Abstract])) OR (Percutaneous Coronary Revascularization[Title/Abstract])) OR (Coronary Revascularization, Percutaneous[Title/Abstract])) OR (Coronary Revascularizations, Percutaneous[Title/Abstract])) OR (Percutaneous Coronary Revascularizations[Title/Abstract])) OR (Revascularization, Percutaneous Coronary[Title/Abstract])) OR (Revascularizations, Percutaneous Coronary[Title/Abstract]))
#3	(“Medicine, Chinese Traditional”[Mesh]) OR ((Traditional Chinese Medicine[Title/Abstract]) OR (TCM[Title/Abstract]))
#4	(“Acupuncture, Ear”[Mesh]) OR (((((((Acupunctures, Ear[Title/Abstract]) OR (Ear Acupunctures[Title/Abstract])) OR (Auricular Acupuncture[Title/Abstract])) OR (Ear Acupuncture[Title/Abstract])) OR (Acupuncture, Auricular[Title/Abstract])) OR (Acupunctures, Auricular[Title/Abstract])) OR (Auricular Acupunctures[Title/Abstract]))
#5	(RCT[Title/Abstract]) OR (randomized controlled trial[Title/Abstract])
#6	#1 and #2 and #3 and #4 and #5

### 2.5 Data collection and analysis

#### 2.5.1 Selection of studies

**Figure 1:**
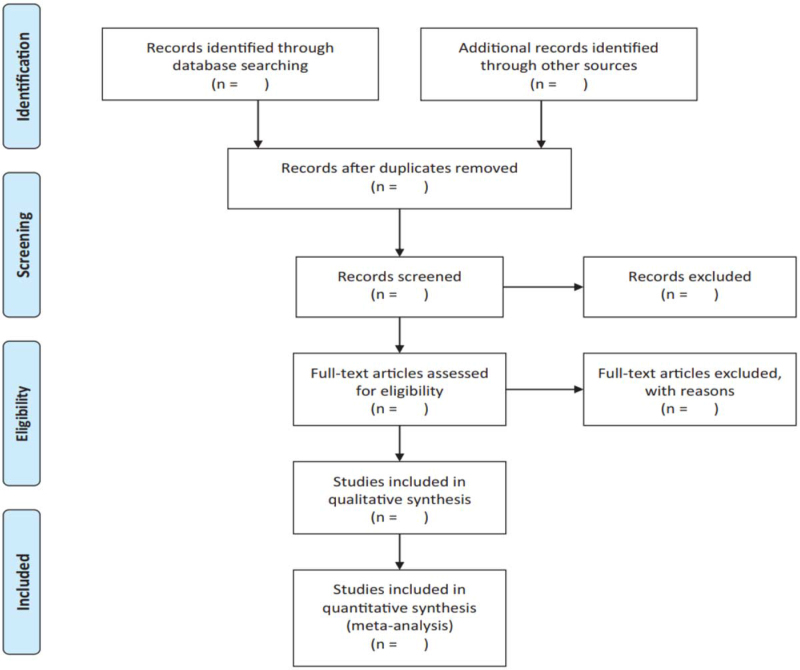
The PRISMA flow chart of the selection process. PRISMA = preferred reporting items for systematic reviews and meta-analyses.

The basic process of including literature will be pursued in reference to the Cochrane Collaboration System Evaluator Manual (5.1.0). Relevant literatures will be obtained from specified databases, later imported into an Endnote X9 software (Camelot UK Bidco Limited, London, United Kingdom) created database. Duplicate documents will be screened out through this process. Independent screening of titles, abstracts, and keywords of all retrieved records will be performed by 2 researchers. The name of the study, author, publishing year, country, database, and justification of the study meeting eligibility criteria to be therefore included in the review will be documented within an excel spreadsheet. Reasons of inclusion and exclusion (participants interventions comparison outcome study design) are disclosed in a spreadsheet during abstract screening and full-text evaluation. A third researcher will be required on making the final decision to resolve any disagreement among the 2 researchers on literatures. The screening flow diagrams of this study will be shown in Figure 1.

#### 2.5.2 Data extraction and management

Two independent reviewers will extract the data of interest from the eligible study and fill in the data collection sheet. If consensus on data extraction is failed to reach by discussion, the decision will be made by the third reviewer. Microsoft Excel (Microsoft corporation) 2013 will be used for data and information management. We will extract the following data:

1. The basic characteristics of RCT: title, 1st author, publishing year, country, and the journal.
2. Participants’ characteristics: average age, gender, sample size, inclusion and exclusion criteria, baseline situation, type, and criteria for the classification of depression.
3. Interventions: treatment duration, study design, randomization, allocation concealment, and blinding methods.
4. Comparators: western medicines.
5. Outcomes: measures, primary and secondary outcomes, security indexes, and follow-up.

#### 2.5.3 Assessment of risk of bias

Cochrane bias risk tool (RevMan 5.3.5; The Nordic Cochrane Centre, The Cochrane Collaboration, Copenhagen, Denmark) will be employed to evaluate the risk of bias, while the following 6 domains will be assessed: random sequence generation, allocation concealment, blinding, incomplete outcome data, selective reporting, and other bias. Each potential trial of bias will be graded as high, low, and unclear. When the 2 independent reviewers failed to reach a consensus on the risk of bias assessment by negotiation, a third reviewer will make a final decision.

#### 2.5.4 Measures of treatment effect

Mean differences (MD) or standard MD with 95% confidence intervals (CIs) will be used as continuous data, and the dichotomous outcomes will be estimated by the risk ratio with 95% CIs.

#### 2.5.5 Unit of analysis issues

Only the 1st experimental period data of crossover trials will be extracted in order to minimize carryover effects. For trials regarding multiple interventions, all relevant experimental groups and control groups within the trial will be combined into a single group to avoid unit-of-analysis error.

#### 2.5.6 Management of missing data

For missing data, we will first try to contact the original author. The research would be excluded from the study if the data failed to be provided on request.

#### 2.5.7 Assessment of heterogeneity

Visual inspection of the forest plots and standard χ^2^ test and Ι^2^ test will be employed to assess heterogeneity. When *P* > .1, Ι^2^ < 50%, it will be considered as no significant heterogeneity between the trials, and the fixed effect model will be applied for statistics, otherwise, the random effect model will be chosen. When heterogeneity occurs, sensitivity analysis or meta-regression will be performed to assess the source of heterogeneity.

#### 2.5.8 Assessment of reporting biases

If 10 or more studies are included in the meta-analysis, funnel plots and Egger test will be used to evaluate the reporting bias. The trim and fill method will be applied to identify and correct asymmetric funnel arising from publication bias, if appropriate.^[16]^

#### 2.5.9 Data synthesis

Data analysis and synthesis will be performed using RevMan version 5.3 software provided by the Cochrane Collaboration. The software will be used to obtain forest plots and test the heterogeneity between the included studies. Risk ratio with 95% CIs will be used for dichotomous data, while the continuous data will be analyzed by MD or standard MD with 95% CIs. Heterogeneity will be assessed by visual inspection of the forest plots and detected by standard χ^2^ test and I^2^ test. When *P* > .1, I^2^ < 50%, it will be considered as no significant heterogeneity between the trials, and the fixed effect model will be applied for statistics, otherwise, the random effect model will be chosen. When heterogeneity occurs, sensitivity analysis or meta-regression will be performed to assess the source of heterogeneity.

#### 2.5.10 Subgroup analysis

When heterogeneity is detected, subgroup analysis will be used (eg, different types of western medicines therapies, patient conditions, research quality, publication age, and participation population) to spot the source of heterogeneity.

#### 2.5.11 Sensitivity analysis

In trials with sufficient data, sensitivity analyses will be taken to test the robustness and reliability of the results. Our sensitivity analysis will be based on heterogeneity, sensitivity analysis may be performed, and certain low-quality or unblinded studies would be excluded when heterogeneity occurs.

## 3 Discussion

PCI is the treatment of choice for many patients with significant coronary artery disease. More than 400,000 procedures are now performed annually in China.^[3]^ High incidence of post-PCI depression would not only corrupt the physical and mental health of patients, but also threaten the prognosis of post-PCI.^[3]^ A study which included 400 post-PCI depression patients indicated postprocedure depression is an independent predictor of 3-year MACE, mortality, and repeat revascularization.^[17]^ Therefore, active treatment and prevention of depression has very important clinical significance. Pharmacotherapy and psychotherapy are the general treatments for post-PCI depression, but neither is ideal. Pharmacotherapy retained potential adverse effects. Psychotherapy is difficult to popularize because of the high cost of treatment and professional requirements demanded on healthcare personnel. Previous meta-analysis has demonstrated that acupuncture is a safe and effective treatment for depressive disorders.^[18]^ Auricular acupuncture is an adjuvant therapy to the regular acupuncture treatment. It has been used as a therapeutic approach in China since the Han dynasty, and a modern version of the technique was developed at the end of the 1950s,^[19]^ which consists of a method for diagnosis and treatment of physical and psychosomatic disorders by stimulating specific areas of the ear.^[20]^ The effects of the intervention have been explained by neurophysiology and reflexology.^[20]^ However, the safety and efficacy of auricular acupuncture treatment on post-PCI depression remains unclear. This systematic review and meta-analysis will provide a convincing conclusion to justify the efficacy and safety of auricular acupuncture for post-PCI patients. This systematic review will evaluate published RCTs evidence for the effectiveness and safety of auricular acupuncture for post-PCI depression. The conclusion drawn from this review is anticipated to assist clinicians on the treatments of post-PCI depression and benefits corresponding patients.

## Author contributions

**Conceptualization:** Ruozhu Lu, Rui Shi.

**Data curation:** Wen Xue, Qian Guo, Cheng Wang.

**Formal analysis:** Ruozhu Lu, Rui Shi, Miao Zhang.

**Investigation:** Xiao Shao.

**Methodology:** Ruozhu Lu, Rui Shi.

**Project administration:** Rui Shi, Cheng Wang.

**Resources:** Ruozhu Lu, Rui Shi, Wen Xue, Qian Guo.

**Software:** Ruozhu Lu, Qian Guo.

**Supervision:** Yue Deng.

**Validation:** Rui Shi, Xiao Shao.

**Visualization:** Yue Deng.

**Writing – original draft:** Ruozhu Lu.

**Writing – review & editing:** Yue Deng.

## Correction

Yeng Due's name was originally misspelled as Due Yeng. This has been corrected.
